# High cut-off membrane for in-vivo dialysis of free plasma hemoglobin in a patient with massive hemolysis

**DOI:** 10.1186/s12882-018-1051-x

**Published:** 2018-10-04

**Authors:** David Cucchiari, Enric Reverter, Miquel Blasco, Alicia Molina-Andujar, Adriá Carpio, Miquel Sanz, Angels Escorsell, Javier Fernández, Esteban Poch

**Affiliations:** 1 Nephrology and Renal Transplant Unit, Hospital Clínic, Carrer Villaroel 170, 08036 Barcelona, Spain; 2 Liver Intensive Care Unit, Hepatology. Hospital Clínic, Barcelona, Spain

**Keywords:** Free hemoglobin, High-cut off filter, Acute kidney injury, Sepsis, Continuous renal replacement therapy

## Abstract

**Background:**

The possibility of clearing Cell-free Plasma Hemoglobin (CPH) from human plasma may appear attractive, especially when considering the noxious effects that CPH has on the immune function and the renal damage caused by its filtration. The existence of the so-called High Cut-Off (HCO) filters, possessing pores as big as 60 kDa, could potentially allow the clearance of the αβ dimers (31.3 kDa), the form in which the α2β2 hemoglobin tetramers (62.6 kDa) physiologically dissociate in plasma. We present herein the first reported case in which such an attempt was made.

**Case presentation:**

The patient was a 51-year-old man with hemolytic crisis due to glucose-6-phosphate dehydrogenase deficiency, further complicated by pigment-induced nephropathy. He underwent a 48-h CVVHD session, in which a HCO filter was used. The Sieving Coefficient (SC) for CPH was initially 0.08 and decreased to 0.02 after 24 h. This unexpected low SC was due to the initial high concentration of CPH (4.24 g/L). At such concentrations, the α2β2 tetramer poorly dissociates into the αβ dimer; but increases exponentially at concentrations lower than 1 g/L.

**Conclusions:**

Clearance of CPH through a HCO filter is technically feasible but its performance markedly relies on the initial concentration of CPH. Critically ill patients with smoldering hemolysis, as it happens during septic shock or ECMO treatment, may benefit the most from the use of this membrane in order to clear CPH.

## Background

The presence of Cell-free Plasma Hemoglobin (CPH) in human plasma derives from the erythrocytes breakdown and depletion of haptoglobin, the plasmatic protein devoted to CPH scavenging. This usually occurs in massive hemolysis, in the context of which haptoglobin-binding capacity is exhausted and CPH is filtered by glomeruli. The glomerular filtration of hemoglobin can lead to pigmented-cast nephropathy, a fearsome complication that may require urgent hemodialysis [1]. Apart from the cases of massive hemolysis, CPH can also be found in acutely ill patients with compromised liver function because of a decreased production of haptoglobin as well as in patients undergoing extracorporeal treatments like hemodialysis and ECMO. In these cases, the CPH concentration is just slightly higher with respect to the physiological levels and is unable to cause significant kidney damage. However, CPH presents some toxic effects even at low levels, since it alters the pathogen-induced inflammatory response. Animal models have demonstrated that lipopolysaccharide (LPS)-induced release of TNF-α is increased after exposure to CPH [2, 3]. Moreover, a clinical study clearly demonstrated that CPH is a strong predictor of mortality in septic patients [4]. Therefore, the possibility to dialyze CPH in septic patients may represent an intriguing topic of research in the next future. Recently, an in-vitro proof-of-concept model demonstrated that a High-Cut Off (HCO) hemofilter with pores of 60 kDa diameter is able to dialyze the αβ hemoglobin dimer, whose molecular weight is 31.3 kDa [5]. The dimer is that part of the hemoglobin molecule that is actually filtered by the glomerulus and that eventually causes pigmented-cast nephropathy [6]. The tetramer composed by two α and two β subunits, which is the physiological carrier of oxygen within erythrocytes, weighs instead 62.6 kDa; this is scarcely filtered by the glomerulus and by HCO filters. Thus, clearance of CPH depends on the dissociation degree of Hb tetramers into dimers. To the best of our knowledge, we report the first case of a patient with massive hemolysis and Acute Kidney Injury (AKI) treated with a HCO filter that allowed us to verify the possibility to clear CPH in-vivo from human plasma.

## Case presentation

A 51-year-old man presented to the emergency department with fever, abdominal pain and jaundice. Past and recent medical history were unremarkable and the patient did not report any recent trip, risky sexual behaviour, parenteral drug intake or ingestion of potentially contaminated food. The physical examination revealed right hypochondrium and epigastric tenderness and no signs of peritonitis. Lab tests showed Aspartate AminoTransferase (AST) 3560 UI/L, Alanine AminoTransferase (ALT) 4513 UI/L, hyperbilirubinemia (total 16 mg/dL), alkaline phosphatase, GGT 90/418 UI/L, PT 50%, normal pancreatic enzymes and normal renal function. Abdominal ultrasound showed no alterations.

Twenty-four hours after the admission, liver function rapidly declined, with PT 40% and maximum total bilirubin of 47 mg/dL. In parallel, blood test showed an elevation of LDH, haptoglobin consumption, reticulocytosis and AKI stage 3 with creatinine of 4 mg/dL and a peripheral blood smear suggestive of hemolysis. Urinalysis was positive for bilirubin and hemoglobin, while the urinary sediment discarded the presence of red blood cells. Serological tests were positive for IgM Hepatitis A Virus (HAV). In addition, a previously unknown complete glucose-6-phosphate dehydrogenase deficiency was detected.

**Fig. 1:**
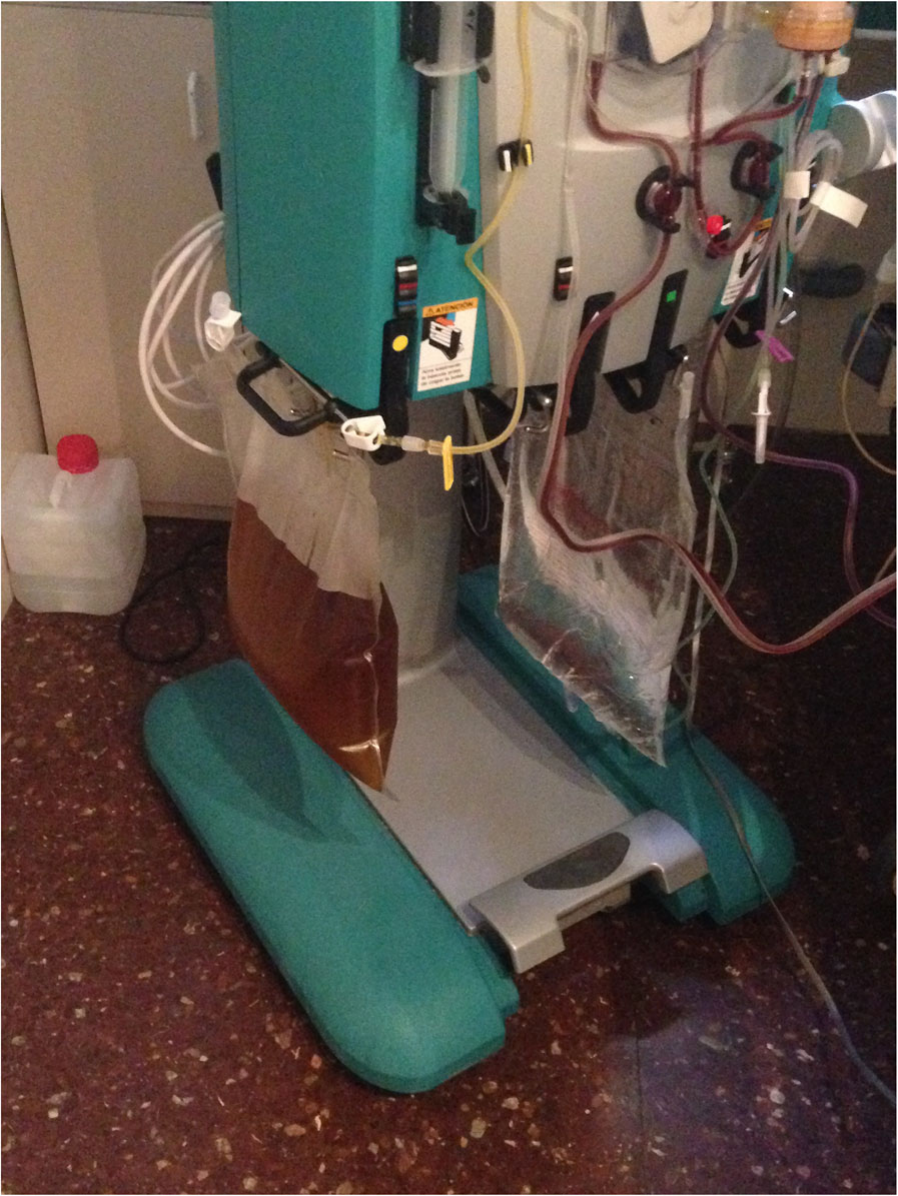
Effluent bag during the first day of therapy. The brownish hue depends on the presence of high bilirubin levels (yellow) along with the filtered CPH (red)

On the basis of these findings a diagnosis of acute hepatitis A infection complicated with massive hemolysis due to glucose-6-phosfate dehydrogenase deficiency was done. Hemolysis was probably triggered by fitomenadione administration and its diagnosis was partially masked by high bilirubin levels due to the severe hepatitis. AKI was interpreted as the result of pigmented-cast nephropathy. The haemolytic crisis was initially managed with 2 sessions of plasma exchange. However, considering the need of dialysis and the presence of CPH, continuous renal replacement treatment with a HCO filter (Septex™, 1.1m^2^, Gambro-Baxter, Hechingen, Germany; Fig. 1) in CVVHD modality was started. In order to measure the CPH clearance, plasma was collected in EDTA tubes from arterial, venous and dialysate ports of the CRRT circuit and CPH was measured with Drabkin-based spectrophotometric analysis at 540 nm. The Sieving coefficient (SC) was calculated as C_D_/[C_In_ + C_Out_)/2] where C_D_, C_In_ and C_Out_ represent CPH concentration at the dialysate, blood inlet and blood outlet side. CPH clearance was calculated as {C_D_/[C_In_ + C_Out_)/(2)]}Q_e_, where Q_e_ is the effluent flow. This filter was used for 48 h, observing a CPH value of 4,24 g/L at the beginning and of 3,72 g/L at the end of the treatment. The calculated SC for CPH was 0.08 at treatment start, later decreasing to 0.02 after 24 h. The calculated clearance of CPH declined as well, from 2,87 ml/min on the first day to 0,76 ml/min after 24 h (Table 1 for treatment data and CPH clearance profile, Table 2 for common laboratory data before and after CVVHD treatment).

**Table 1:** Treatment data and CPH concentrations 30′, 24 h and 48 h after CRRT start

	30 min	24 h	48 h
C_In_ (g/L)	4,24	4,33	3,72
C_Out_ (g/L)	4,23	4,19	3,66
C_D_ (g/L)	0,37	0,1	0,07
Sieving Coefficient	0,087	0,023	0,018
Clearance (ml/min)	2,87	0,76	0,62
Q_b_ (ml/min)	250	250	250
Q_d_ (ml/min)	33	33	33
Q_e_ (ml/Kg/h)	28,2	29	29
UF (ml/h)	0	50	50

*C*_*In*_ CPH concentration at the arterial side, *C*_*Out*_ CPH concentration at the venous side, *C*_*D*_ CPH concentration at dialysate side, *Q*_*b*_ blood flow, *Q*_*d*_ dialysate flow, *Q*_*e*_ effluent flow, *UF* UltraFiltration rate

**Table 2:** Main laboratory values before and after 48-h long treatment with the HCO membrane

	Before HCO-CVVHD	After HCO-CVVHD
Creatinine (mg/dL)	6.48	3.55
AST/ALT (UI/L)	196/314	106/208
Alkaline Phosphatase (UI/L)	66	89
Gamma-GT (UI/L)	51	111
Direct Bilirubine (mg/dL)	31.2	24.2
Indirect Bilirubine (mg/dL)	4.8	4
Haptoglobine (g/L)	Undetectable	0.54
C-Reactive Protein (mg/dL)	6.48	5.89
Na/K (mEq/L)	140/4.4	137/4
Albumin (g/L)	28	28
Total protein (g/L)	41	50
Hemoglobin (g/L)	81	108
Platelets (× 10^3^/L)	192	177
White Blood Cells (×10^3^/L)	13.53	7.29
Prothrombin Time (%)	69.7	59.9

After this treatment, as the patient was still oliguric, seven sessions more of intermittent hemodialysis were performed. Eventually, 4 weeks after admission urine output ensued and the patient’s renal function started to recover. At the time of discharge, total bilirubin was 3.4 mg/dL and creatinine 2.8 mg/dL. Finally, 3 months after discharge the patient presented normal renal and hepatic function.

## Discussion and conclusions

To the best of our knowledge, we report the first case of in-vivo CPH clearance by means of HCO-based CVVHD treatment. HCO filters have been designed to improve clearance of middle-sized molecules that were not previously cleared by normal filters, such as pro- and anti-inflammatory cytokines [7–10]. The possibility to clear CPH has been recently examined in an in-vitro model, in which the authors demonstrated the efficacy of the same HCO filter that we used, with a SC for CPH of 0.35 and a clearance of approximately 22 ml/min [5]. However, in our case the initial SC was of only 0.08, decreasing to 0.02 in the following 24 h, presumably due to the phenomenon of protein-coating. CPH clearance was reduced as well, with values below 3 ml/min initially: albeit not negative, this clearance is far from being clinically relevant. In order to explain the striking difference with the results obtained in the in-vitro model, we should consider the dissociation characteristics of the hemoglobin tetramers (62.6 kDa) into dimers (31.3 kDa). Indeed, the hemoglobin dimer is the part of the molecule that is cleared by the HCO filter under study, whose cut-off is 60 kDa. As the dissociation of tetramers into dimers is maximum at low concentrations of CPH, the best results are obtained with CPH concentrations lower than 1 g/L. In the above-cited in-vitro study, indeed, the CPH concentration was within this range and the approximate dissociation degree at this concentration was around 40%. On the other side, our patient had much higher CPH concentration (4.24 g/L). At this level, the expected dissociation degree is lower than 10% [5]. Therefore, the lower SC for CPH observed in our case is probably due to the lesser degree of dissociation of the molecule. This point provides an important indication, since it demonstrates that those patients that would benefit the most from this treatment are those presenting CPH levels lower than 1 g/L. Presumably, patients with massive hemolysis and already established kidney injury are less likely to benefit from HCO-based CPH clearance. On the other side, critically ill patients with smoldering hemolysis and lower concentrations of CPH should theoretically take more advantage from it. For example, pediatric patients submitted to two different types of ECMO treatment had mean CPH values between 0.36 and 0.58 g/l [11], while CPH values in septic patients ranged from 0.034 to 0.185 g/L [4]. It is worthy to underline that even these low levels of CPH are not harmless. In a rat model, purified hemoglobin enhanced TNF-α synthesis in LPS-stimulated macrophages by a factor of 1.000 [2]. In another model, the administration of hemoglobin in LPS-treated mice resulted in an increase in TNF-α levels and mortality [3]. In humans, CPH was strongly associated with decreased survival in severe septic patients, even after substantial correction with already known risk factors including age, inflammatory biomarkers, Simplified Acute Physiology (SAPS-II) and Sequential Organ Failure Assessment (SOFA) scores [4].

## Conclusion

In conclusion, we report the case of a patient treated with a HCO-based CRRT treatment for the clearance of CPH. The lower than expected SC was probably due to the low dissociation of the hemoglobin tetramers into dimers, as a consequence of the high concentrations of CPH in our patient. Therefore, we might infer that those patients that would benefit the most from this kind of treatment are the critically ill ones with low levels of CPH in the context of septic shock or extracorporeal treatments like ECMO. Even at these low levels, CPH has well-documented harmful effects on the immune system. The possibility to clear CPH in these patients may not represent only a “cosmetic” intervention, but may also be beneficial in blunting the inflammatory response.

## Abbreviations

AKI: Acute Kidney Injury
ALT: Alanine AminoTransferase
AST: Aspartate AminoTransferase
CRRT: Continuous Renal Replacement Therapy
CVVHD: Continuous Veno-Venous HemoDialysis
ECMO: Extracorporeal Membrane Oxygenation
HAV: Hepatitis A Virus
HCO: High-Cut Off
LPS: lipopolysaccharide
SAPS-II: Simplified Acute Physiology Score II
SOFA: Sequential Organ Failure Assessment
TNF-α: Tumor Necrosis Factor Alfa
